# Supplementary material for: Dynalign II: common secondary structure prediction for RNA homologs with domain insertions
Source: Nucleic Acids Res. 2014 Nov 21;42(22):13939–48. doi: 10.1093/nar/gku1172 (PMC4267632; doi:10.1093/nar/gku1172)
Supplement: SUPPLEMENTARY DATA [file supp_gku1172_nar-02021-z-2014-File012.zip › manual/RNA_class/html/class_dynalign__object-members.html]

RNAstructure Classes: Member List

- Main Page
- Classes
- Files

- Class List
- Class Hierarchy
- Class Members

# Dynalign\_object Member List

This is the complete list of members for Dynalign\_object, including all inherited members.

|  |  |  |
| --- | --- | --- |
| align | Dynalign\_object | `[private]` |
| AllocateForceAlign() | Dynalign\_object | `[private]` |
| array | Dynalign\_object | `[private]` |
| CommonConstructor() | Dynalign\_object | `[private]` |
| compoundmessage | TwoRNA |  |
| ct\_templated | Dynalign\_object | `[private]` |
| data | Dynalign\_object | `[private]` |
| dsv\_templated | Dynalign\_object | `[private]` |
| Dynalign(const short int maxtrace, const short int bpwin, const short int awin, const short int percent, const short int imaxseparation=-99, const float gap=0.4, const bool singleinsert=true, const char savefile[]=NULL, const bool optimalonly=false, const short int singlefold\_subopt\_percent=30, const bool local=false, const short int numProcessors=1, const int maxpairs=-1) | Dynalign\_object |  |
| Dynalign\_object() | Dynalign\_object |  |
| Dynalign\_object(const char sequence1[], const char sequence2[], const bool IsRNA=true) | Dynalign\_object |  |
| Dynalign\_object(const char filename1[], const int type1, const char filename2[], const int type2, const bool IsRNA=true) | Dynalign\_object |  |
| Dynalign\_object(const char filename[]) | Dynalign\_object |  |
| Dynalign\_object(const char \*filename, const short maxtrace, const short bpwin, const short awin, const short percent) | Dynalign\_object |  |
| ErrorCodeTwo | TwoRNA | `[protected]` |
| forcealign | Dynalign\_object | `[private]` |
| ForceAlignment(const int i, const int k) | Dynalign\_object |  |
| gap | Dynalign\_object | `[private]` |
| GetBestPairEnergy(const int sequence, const int i, const int j) | Dynalign\_object |  |
| GetErrorCode() | TwoRNA |  |
| GetErrorMessage(const int error) | Dynalign\_object |  |
| GetErrorMessageString(const int error) | TwoRNA |  |
| GetForcedAlignment(const int i, const int seq) | Dynalign\_object |  |
| GetLowestEnergy() | Dynalign\_object |  |
| GetRNA1() | TwoRNA |  |
| GetRNA2() | TwoRNA |  |
| GetTemperature() | TwoRNA |  |
| highend | Dynalign\_object | `[private]` |
| lowend | Dynalign\_object | `[private]` |
| lowest | Dynalign\_object | `[private]` |
| MAXDSV | Dynalign\_object | `[private]` |
| Maxtrace | Dynalign\_object | `[private]` |
| modificationflag | Dynalign\_object | `[private]` |
| ReadAlignmentConstraints(const char filename[]) | Dynalign\_object |  |
| savefileread | Dynalign\_object | `[private]` |
| SetProgress(TProgressDialog &Progress) | Dynalign\_object |  |
| SetTemperature(double temperature) | TwoRNA |  |
| StopProgress() | Dynalign\_object |  |
| storetemplatefilename(const char \*name) | Dynalign\_object | `[private]` |
| templatefilename | Dynalign\_object | `[private]` |
| Templatefromct(const char ctfilename[]) | Dynalign\_object |  |
| Templatefromdsv(const char dsvfilename[], const float maxdsvchange) | Dynalign\_object |  |
| TwoRNA(const char sequence1[], const char sequence2[], bool IsRNA=true) | TwoRNA |  |
| TwoRNA(const char filename1[], const int type1, const char filename2[], const int type2, bool IsRNA=true) | TwoRNA |  |
| TwoRNA() | TwoRNA |  |
| v | Dynalign\_object | `[private]` |
| vmod | Dynalign\_object | `[private]` |
| w | Dynalign\_object | `[private]` |
| w3 | Dynalign\_object | `[private]` |
| w5 | Dynalign\_object | `[private]` |
| WriteAlignment(const char filename[]) | Dynalign\_object |  |
| ~Dynalign\_object() | Dynalign\_object |  |
| ~TwoRNA() | TwoRNA |  |

---

Generated on Tue Jul 30 10:52:29 2013 for RNAstructure Classes by 
 1.5.7.1
